# Solid serous adenoma of the pancreas mimicking a solid pseudopapillary neoplasm: A case report

**DOI:** 10.1097/MD.0000000000049671

**Published:** 2026-07-10

**Authors:** Sohui Jin, Jisun Lee, Bum Sang Cho, Kyung Sik Yi, Yook Kim, Chi-Hoon Choi, Chang Gok Woo

**Affiliations:** a Department of Radiology, Chungbuk National University Hospital, Cheongju, Chungcheongbuk-do, Republic of Korea; b Department of Radiology, College of Medicine, Chungbuk National University, Chungbuk National University Hospital, Cheongju, Chungcheongbuk-do, Republic of Korea; c Department of Pathology, College of Medicine, Chungbuk National University, Chungbuk National University Hospital, Cheongju, Chungcheongbuk-do, Republic of Korea.

**Keywords:** case report, multimodality imaging, pancreas, serous cystic neoplasm, solid serous adenoma

## Abstract

**Rationale:**

Serous cystic neoplasms of the pancreas are benign epithelial tumors that typically present as microcystic lesions. However, a rare solid variant, known as solid serous adenoma, may appear as a solid hypervascular mass and mimic other solid pancreatic tumors.

**Patient concerns:**

An asymptomatic woman in her early 50s was referred for evaluation of an incidentally detected pancreatic tail mass.

**Diagnoses:**

Computed tomography and magnetic resonance imaging demonstrated heterogeneous peripheral enhancement with progressive centripetal fill-in and a persistent hypoenhancing area. Based on these findings, a solid pseudopapillary neoplasm was initially suspected.

**Interventions:**

Because malignancy could not be excluded on imaging, the patient underwent distal pancreatectomy.

**Outcomes:**

Histopathological examination confirmed solid serous adenoma composed of glycogen-rich cuboidal epithelium forming numerous microcystic spaces. The imaging findings correlated with regional differences in stromal composition. The postoperative course was uneventful, and follow-up imaging showed no evidence of recurrence or residual disease.

**Lessons:**

Solid serous adenomas can exhibit variable imaging appearances and mimic other solid pancreatic tumors. Recognition of this entity is clinically important, as it may improve diagnostic accuracy and help prevent unnecessary surgical resection of benign pancreatic lesions.

## 1. Introduction

Serous cystic neoplasms (SCNs) of the pancreas are benign epithelial tumors composed of glycogen-rich cuboidal cells that form multiple cystic spaces.^[1–3]^ According to the World Health Organization classification, SCNs represent a spectrum of lesions characterized by their cystic architecture and benign biological behavior.^[3,4]^ Although most SCNs demonstrate a characteristic microcystic appearance on imaging, a rare solid variant, commonly referred to as solid serous adenoma, may present as a hypervascular solid mass because its microscopic cystic spaces are not discernible on routine cross-sectional imaging.^[5,6]^ This atypical imaging presentation poses a diagnostic challenge, since solid SCNs frequently overlap with other solid pancreatic neoplasms. Among these, pancreatic neuroendocrine tumors (PanNETs) are the most commonly presumed diagnosis in reported cases.^[7,8]^ In less typical cases, imaging overlap with solid pseudopapillary neoplasms (SPNs) or other solid tumors may also occur.^[5,9]^

Accurate preoperative differentiation is clinically important because SCNs are benign lesions that may be managed conservatively, whereas their principal differential diagnoses often require surgical resection.^[10,11]^ However, due to overlapping imaging features and the absence of specific diagnostic criteria, a definitive diagnosis remains difficult in many cases, and misdiagnosis may lead to unnecessary surgical resection of a benign lesion. In this report, we describe a case of solid serous adenoma with an unusual progressive enhancement pattern mimicking an SPN, an uncommon diagnostic pitfall. We further describe the radiologic–pathologic correlation underlying these imaging findings, with emphasis on its clinical relevance in avoiding misdiagnosis of benign pancreatic lesions.

## 2. Case presentation

A woman in her early 50s was referred to our hospital for evaluation of an incidentally detected suprarenal mass identified on abdominal ultrasonography during a routine health checkup at an outside clinic. Based on its location on ultrasonography, the lesion was initially suspected to originate from the left adrenal gland. At presentation, the patient was asymptomatic, and her vital signs along with physical examination findings were all within normal limits. Her medical history was notable for morbid obesity (body mass index, 37.2 kg/m^2^) and a hysterectomy with bilateral salpingo-oophorectomy performed 10 years earlier. She had no other significant medical history and was not taking any regular medications. Initial laboratory test results, including tumor markers such as carbohydrate antigen 19-9, cancer antigen 125, and carcinoembryonic antigen, were within normal limits.

Transabdominal ultrasonography performed at the outside institution revealed a well-defined ovoid mass with heterogeneous echogenicity, measuring approximately 6.7 cm, in the left suprarenal region. Color Doppler imaging showed mild peripheral vascularity. Based on its location and ultrasonographic appearance, an adrenal mass was initially suspected. Because the lesion was large and the possibility of a solid pancreatic neoplasm with malignant potential could not be excluded, further imaging evaluation was performed.

**Figure 1.**
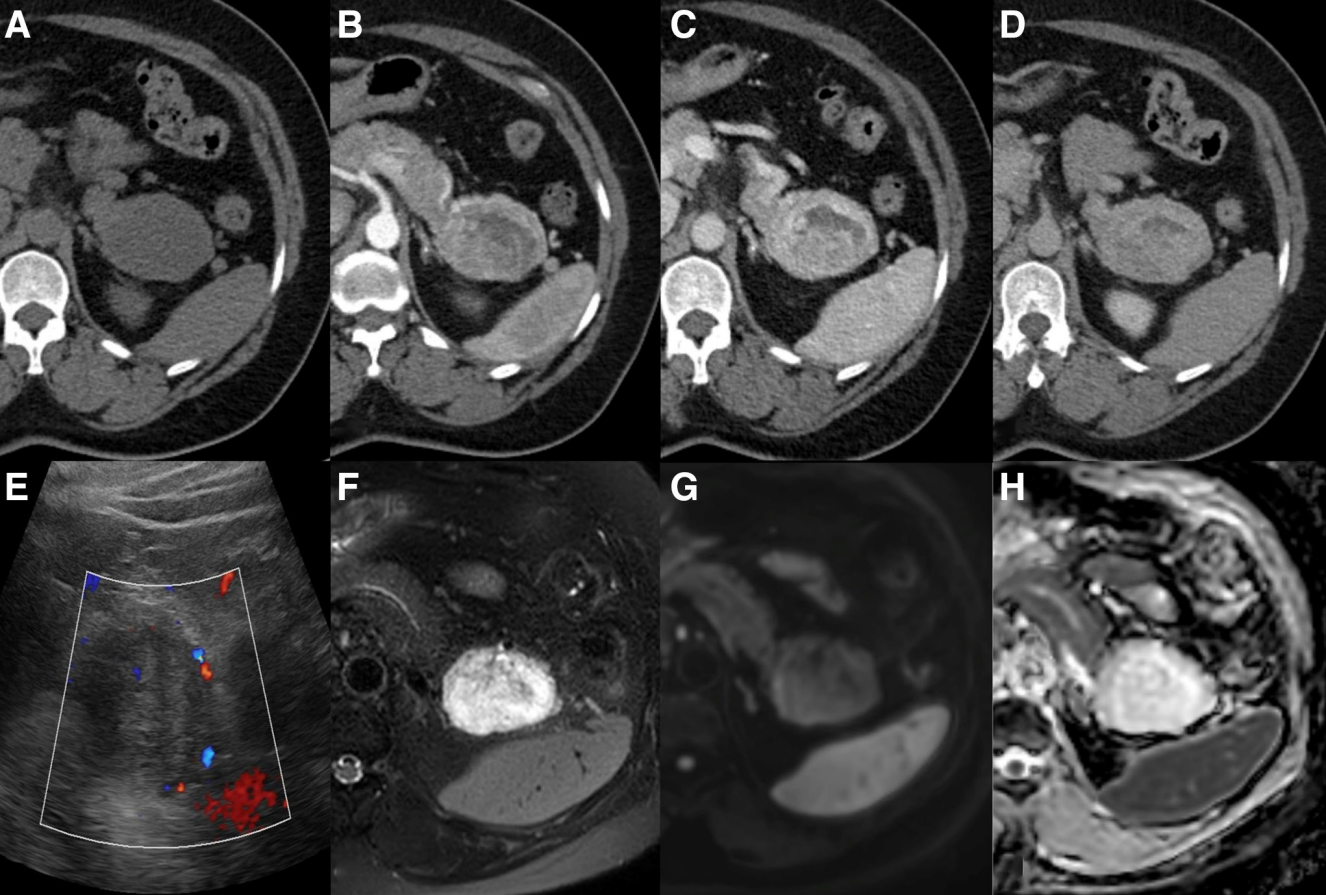
Multimodality imaging findings of a solid serous adenoma in the pancreatic tail. (A) Precontrast CT image shows lower attenuation of the lesion than of the adjacent pancreatic parenchyma. (B) Arterial-phase CT demonstrates heterogeneous peripheral enhancement. (C) Portal venous-phase CT shows progressive centripetal fill-in. (D) Delayed-phase CT image shows gradual enhancement of the central portion, whereas a crescent-shaped region remains persistently hypoattenuating. (E) Transabdominal ultrasonography demonstrates a well-defined ovoid mass with heterogeneous echogenicity and mild peripheral vascularity on color Doppler imaging. (F) T2-weighted MR image shows heterogeneous hyperintensity. (G) Diffusion-weighted image shows mildly increased signal intensity. (H) Apparent diffusion coefficient map shows no corresponding low signal intensity, indicating the absence of true diffusion restriction. CT = computed tomography, MR = magnetic resonance.

Abdominal computed tomography (CT) was subsequently performed to clarify the origin of the mass and revealed a well-defined 5.6 cm lesion arising from the pancreatic tail (Fig. 1). On precontrast imaging, the lesion showed lower attenuation (19.4 Hounsfield unit) than the adjacent pancreatic parenchyma (40.9 Hounsfield unit), corresponding to an attenuation value ratio (AVR) of 0.47 (tumor-to-parenchyma attenuation ratio). No internal calcification or hemorrhage was observed. During arterial-phase imaging, the mass showed heterogeneous peripheral enhancement, whereas the central portion was hypoattenuating. On portal venous-phase imaging, the mass demonstrated progressive centripetal enhancement. On delayed images, the central portion showed gradual enhancement; however, a crescent-shaped region remained persistently hypoattenuating.

Contrast-enhanced pancreatic magnetic resonance imaging (MRI) was performed for further characterization. The mass was hypointense on T1-weighted images and heterogeneously hyperintense on T2-weighted images. Diffusion-weighted imaging showed high signal intensity without convincing diffusion restriction, with elevated apparent diffusion coefficient (ADC) values ranging from 2.23 to 2.43 × 10^−^3 mm^2^/s. Dynamic contrast-enhanced MRI demonstrated peripheral enhancement during the arterial phase, followed by gradual centripetal fill-in on subsequent phases. Consistent with CT findings, a crescent-shaped region remained persistently hypoenhancing even on delayed images. A thin peripheral rim with delayed enhancement was also observed.

Based on these imaging features: including a well-circumscribed pancreatic tail mass with heterogeneous internal architecture, T2 hyperintensity, and gradual centripetal enhancement: the leading diagnosis was SPN, with PanNET considered as an additional differential diagnosis. Because the lesion was considered surgically resectable and malignancy could not be excluded on imaging, the patient underwent laparoscopic distal pancreatectomy with splenectomy without preoperative biopsy.

**Figure 2.**
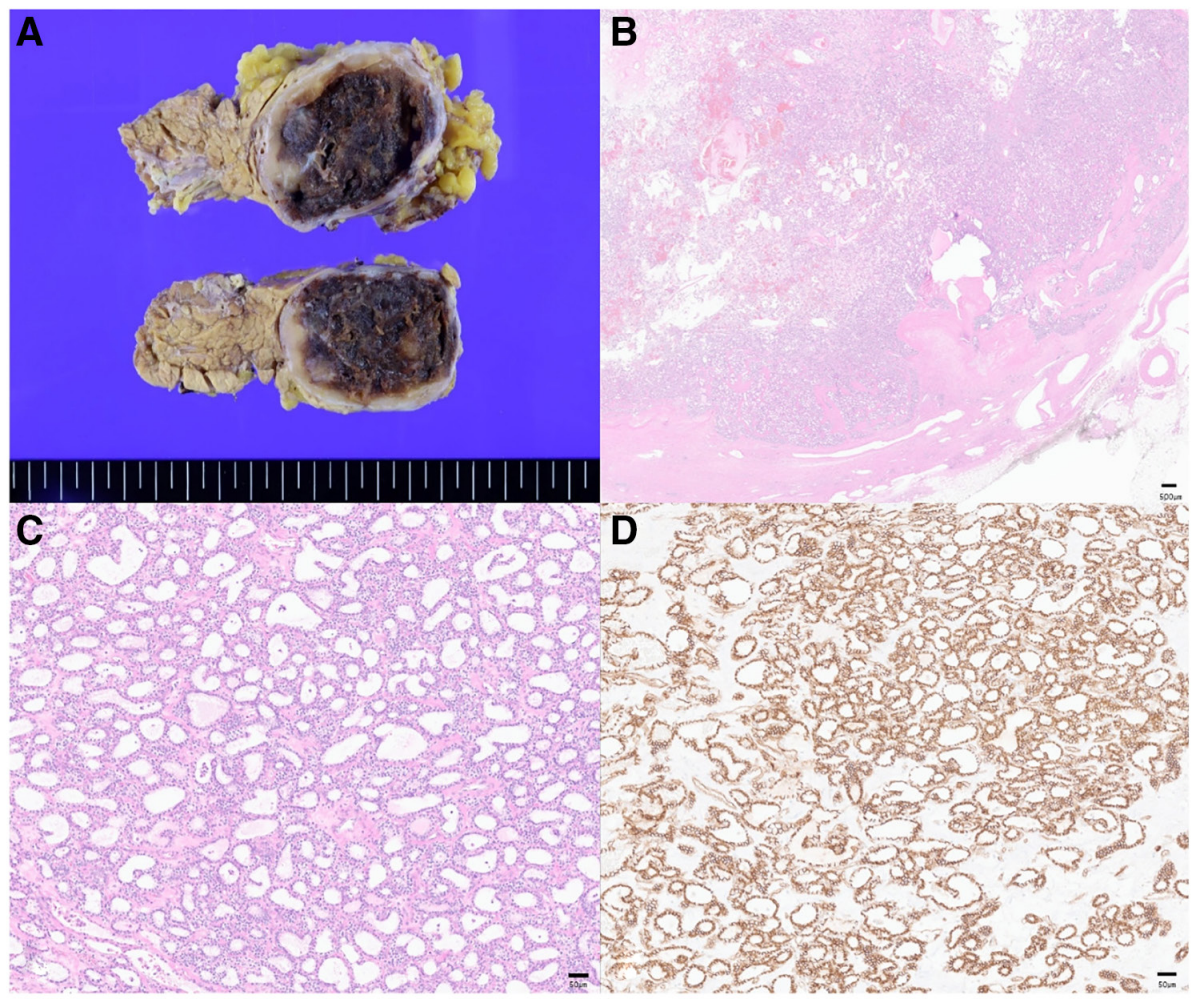
Gross and histopathologic findings of solid serous adenoma. (A) Gross specimen shows a well-circumscribed mass with a heterogeneous cut surface and a fibrous capsule. (B) Low-power histologic image (scale bar 500 µm) demonstrates stromal and microcystic components with hemorrhage. The microcystic components are more densely distributed at the periphery and become progressively sparser toward the center, corresponding to the heterogeneous delayed enhancement pattern. (C) Higher magnification histologic image (scale bar 50 µm) shows numerous tightly packed microcysts lined by a single layer of cuboidal epithelial cells with abundant clear cytoplasm. (D) Immunohistochemical staining (scale bar 50 µm) for β-catenin shows absence of nuclear expression, helping exclude solid pseudopapillary neoplasm.

Gross examination revealed a well-circumscribed pancreatic tumor surrounded by a thick fibrous capsule. The cut surface was reddish-brown and soft, with focal hemorrhage and internal septations. Histologically, the tumor was encapsulated by dense fibrous tissue. At low magnification, cystic spaces and stromal components were more densely distributed at the periphery and became progressively sparser toward the center, with prominent fibrocollagenous trabeculae and focal old hemorrhage. At higher magnification, the tumor consisted of numerous tightly packed microcysts lined by a single layer of cuboidal epithelial cells with abundant clear cytoplasm, separated by hyalinized fibrous septa. Periodic acid-Schiff staining demonstrated strong cytoplasmic positivity that disappeared after diastase digestion, indicating abundant intracytoplasmic glycogen. Immunohistochemical analysis showed absent nuclear β-catenin expression with preserved membranous E-cadherin expression, excluding SPN. Tumor cells were negative for synaptophysin, CD56, and vimentin, arguing against PanNET and metastatic renal cell carcinoma. These findings supported the diagnosis of solid serous adenoma (Fig. 2).

The postoperative course was uneventful, and the patient was discharged on postoperative day 14. Follow-up CT performed 2.5 months later showed no evidence of recurrence or residual disease.

## 3. Discussion

Solid serous adenoma, a solid variant of SCN, is an uncommon presentation that poses a diagnostic challenge because its microscopic cystic architecture is not visible on routine imaging, resulting in a solid-appearing mass.^[5,7]^ Previously reported cases of pancreatic solid serous adenoma are summarized in Table 1. Consequently, these lesions are frequently misinterpreted as true solid pancreatic neoplasms despite their benign nature.^[1,7]^ In the present case, this challenge was further compounded by an atypical enhancement pattern, which led to a preoperative impression of a solid pseudopapillary neoplasm and ultimately to surgical resection of a benign lesion.

**Table 1:** Summary of reported cases of pancreatic solid serous adenoma.

Author	Year	Age	Sex	Symptoms	Tumor Location	Size (cm)	Imaging Modalities	Predominant imaging findings	Preoperative diagnosis	Surgical procedure
Perez-Ordonez et al^[12]^	1996	70	F	Abdominal pain	Tail	4.0	Not stated	Not stated	PanNET	Distal pancreatectomy
Yamamoto et al^[13]^	2004	60	M	Epigastric distention	Uncus	2.0	U, C, M, MC	Uniformly enhancing solid mass	PanNET	Enucleation
Gabata et al^[6]^	2005	59	F	Abdominal pain	Body	2.0	U, C, M, MC	Hypervascular mass with central scar-like enhancement	Solid serous adenoma	Distal pancreatectomy
Yamaguchi^[14]^	2006	60	M	Epigastric distension	Head	2.0	C	Well enhancing mass	PanNET	Whipple procedure
Reese et al^[9]^	2006	66	M	Asymptomatic	Head	3.5	C	Hypervascular mass	PanNET	PPPD
Stern et al^[15]^	2007	62	M	Abdominal pain	Head/Body	4.2	C	Heterogeneously enhancing solid mass	PanNET PDAC SPN	Distal pancreatectomy
Sanaka et al^[16]^	2007	74	M	Asymptomatic	Body	1.5	C, E	Enhancing hypoechoic mass	PanNET	Enucleation + YPJ
Casadei et al^[17]^	2008	59	F	Abdominal pain	Tail	3.5	C	Well-defined, well-enhancing mass	Solid serous adenoma	Distal pancreatectomy
Lam-Himlin et al^[18]^	2010	70	M	Asymptomatic	Tail	6.0	C	Hypervascular mass with calcifications	PanNET	Distal pancreatectomy
Hayashi et al (Case 1)^[19]^	2012	74	F	Not stated	Body	4.2	C	Low attenuation on unenhanced CT, washout, capsule	Not stated	Resection
Hayashi et al (Case 2)^[19]^	2012	57	F	Not stated	Head	2.1	C	Low attenuation on unenhanced CT, washout, capsule	Not stated	Resection
Hayashi et al (Case 3)^[19]^	2012	58	F	Not stated	Body	3.2	C	Low attenuation on unenhanced CT, prolonged enhancement	Not stated	Resection
Kishida et al^[1]^	2014	58	M	Asymptomatic	Body	2.8	U, C, M	Marginal arterial enhancement	PanNET	Distal pancreatectomy
Wu et al (Case 1)^[20]^	2015	48	M	Abdominal cramps	Head	2.7	C, P	Arterial-phase enhancement	NET with metastasis	PPPD
Wu et al (Case 2)^[20]^	2015	65	F	Asymptomatic	Body	2.3	C	Arterial-phase enhancement	PanNET	Distal pancreatectomy
Jang et al^[21]^	2015	62	F	Asymptomatic	Tail	2.4	C, M	Marginal arterial enhancement, high T2 signal intensity	PanNET	Distal pancreatectomy
Katsourakis et al^[22]^	2016	72	F	Abdominal pain	Tail	3.0	M	Well-demarcated lesion with rapid washout	PanNET	Distal pancreatectomy
Okumura et al^[23]^	2018	50	F	Asymptomatic	Body	2.0	C, M, P, MC	Hypervascular solid mass, high signal intensity on MRCP	Solid SCA	Middle segment pancreatectomy
Chen et al (Case 1)^[5]^	2019	69	F	Asymptomatic	Neck	1.5	C, M, MC	Washin/washout with poorly enhancing zone	PanNET	PPPD
Chen et al (Case 2)^[5]^	2019	27	F	Asymptomatic	Tail	3.0	C, M	Washin/washout with poorly enhancing zone	SPN	Distal pancreatectomy
Chen et al (Case 3)^[5]^	2019	49	F	Abdominal pain	Tail	2.0	C, M, MC	Moderate and prolonged enhancement	Pancreatic cystadenoma	Distal pancreatectomy
Chen et al (Case 4)^[5]^	2019	53	F	Asymptomatic	Tail	1.8	C, M, MC	Moderate and prolonged enhancement	PDAC	PPPD
Chen et al (Case 5)^[5]^	2019	23	F	Abdominal distension	Neck	3.2	C, M, MC	Moderate and prolonged enhancement	SPN	PPPD
Demesmaker et al^[24]^	2019	63	M	Weight loss	Isthmus	2.5	C, M, E, P	Hypervascular lesion in the arterial phase	PanNET with liver metastasis	PD
Mohan et al^[25]^	2021	50	F	Abdominal pain	Body	2.5	U, C, P	Arterial hyperenhancement with washout	PanNET	Enucleation
da Costa et al^[8]^	2025	67	F	Abdominal pain	Head	3.5	M	Hypervascular solid mass	PanNET	PD
Present Case	2024	Early 50s	F	Asymptomatic	Tail	5.6	U, C, M	Heterogeneous mass with gradual enhancement	SPN	Distal pancreatectomy

Size refers to the largest dimension reported.

This table summarizes previously published individual case reports and does not include large multi-institutional studies such as those by Fang et al (2022) and Jang et al (2015), which primarily reported cohort-level analyses. Quantitative thresholds described in these studies, such as an attenuation value ratio of < 0.77 and an apparent diffusion coefficient >1.99 × 10^−3^ mm^2^/s, may serve as useful reference points for lesion characterization.

CT/C = computed tomography, E = endoscopic ultrasonography, M = magnetic resonance imaging, MC = magnetic resonance cholangiopancreatography, NET = neuroendocrine tumor, P = positron emission tomography, PanNET = pancreatic neuroendocrine tumor, PD = pancreaticoduodenectomy, PDAC = pancreatic ductal adenocarcinoma, PPPD = pylorus-preserving pancreaticoduodenectomy, SCA = serous cystadenoma, SPN = solid pseudopapillary neoplasm, U = ultrasonography, YPJ = Roux-en-Y pancreaticojejunostomy.

On cross-sectional imaging, solid serous adenoma most often appears as a well-defined hypervascular mass reflecting its rich microvascular network within a fibrous stroma.^[5,19,25]^ Dynamic CT findings are variable and may include arterial-phase hyperenhancement with washin/washout or more prolonged enhancement patterns; however, these features are not specific and frequently overlap with those of other solid pancreatic neoplasms, making preoperative differentiation challenging in clinical practice.^[3,6,8,9,23,25]^ On MRI, relatively high T2 signal intensity may be observed, reflecting the underlying microscopic cystic architecture that is not visible on routine imaging. In contrast to conventional microcystic SCN, in which cystic components are readily discernible, this feature may further complicate preoperative diagnosis.^[6,23]^ Consequently, preoperative differentiation remains challenging in most reported cases, with PanNET representing the most common presumptive diagnosis, often leading to consideration of surgical resection.^[8]^ In contrast to this commonly encountered diagnostic context, the present case exhibited predominantly progressive enhancement with partial centripetal fill-in, accompanied by a persistent crescent-shaped hypoenhancing region on delayed imaging, further contributing to diagnostic uncertainty. This atypical enhancement pattern, together with the pancreatic tail location, heterogeneous internal architecture, and fibrous capsule, led to an imaging impression more suggestive of SPN, a tumor that often requires surgical resection.

Quantitative imaging parameters can offer additional information for differentiating solid serous adenomas from other solid pancreatic tumors. On unenhanced CT, solid serous adenomas typically demonstrate low attenuation, reflecting their fluid-rich, cystic microscopic composition. Fang et al^[10]^ reported that an AVR of < 0.77, together with washin/washout enhancement patterns, may help distinguish these tumors from nonfunctioning PanNETs. In the present case, the precontrast AVR was 0.47; however, the lesion demonstrated predominantly progressive rather than washin/washout enhancement, highlighting the variability and limited specificity of these parameters.

Similarly, diffusion-weighted MRI may contribute to lesion characterization, as solid serous adenomas generally exhibit relatively high ADC values, reflecting low cellularity and abundant cystic components. Jang et al^[21]^ reported that ADC values above 1.99 × 10^−^3 mm^2^/s, together with high T2 signal intensity, may help differentiate these lesions from PanNET. In the present case, ADC values were also relatively high; however, substantial overlap with other solid pancreatic tumors limits the specificity of ADC as a standalone discriminator. Therefore, these quantitative parameters should be interpreted as supportive rather than definitive findings.

**Figure 3.**
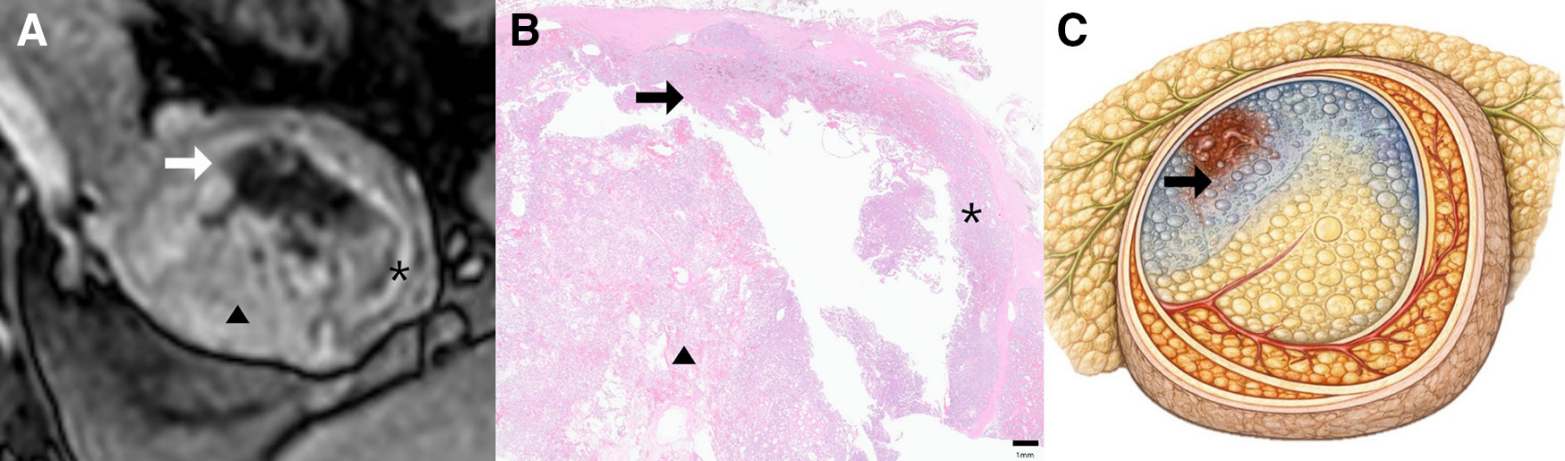
Radiologic–pathologic correlation of the heterogeneous delayed enhancement pattern. (A) Portal-phase contrast-enhanced T1-weighted MR image shows gradual enhancement of the central portion (arrowhead), whereas a crescent-shaped region remains persistently hypoenhancing (arrow). Asterisk indicates the peripherally enhancing portion of the lesion. (B) Low-power histologic image (scale bar 1mm) demonstrates stromal and microcystic components with hemorrhage. The asterisk indicates densely packed peripheral microcysts, the arrowhead denotes relatively sparse central fibrocollagenous tissue, and the arrow marks a crescent-shaped fibrotic area corresponding to the persistently hypoenhancing region on MRI. Focal hemorrhagic change may also contribute to the observed imaging heterogeneity. (C) The schematic illustration summarizes the corresponding regional heterogeneity, including densely packed peripheral microcysts (asterisk), relatively sparse central fibrocollagenous tissue (arrowhead), and a crescent-shaped fibrotic area (arrow), accounting for the atypical imaging appearance. MRI = magnetic resonance imaging.

Beyond the quantitative findings, one of the most distinctive features of the present case was its heterogeneous progressive enhancement pattern, characterized by partial centripetal fill-in and a persistent crescent-shaped hypoenhancing region even on delayed imaging, which contributed to preoperative diagnostic uncertainty. This appearance can be explained by the underlying histopathologic heterogeneity of solid serous adenoma, which consists of innumerable microscopic cysts separated by fibrous septa and supported by variably distributed fibrovascular stroma.^[1,19,25]^ In this case, more densely packed microcystic spaces were observed at the periphery, whereas the central portion showed relatively sparse cystic structures with prominent fibrocollagenous tissue. This regional variation likely accounts for the mixed enhancement pattern, with earlier enhancement of the more vascular peripheral portion and delayed enhancement of the more fibrotic central component, potentially mimicking other solid pancreatic neoplasms. The persistent crescent-shaped hypoenhancing area on delayed images most likely reflects a region with relatively abundant fibrous stroma and reduced microvascularity, corresponding to the fibrotic component observed histologically (Fig. 3). In addition, focal old hemorrhagic change may also contribute to this appearance. This feature may be misinterpreted as a necrotic or nonviable area, thereby raising concern for malignancy on imaging. This radiologic–pathologic correlation helps explain why a benign serous lesion can present as a solid-appearing mass and mimic other pancreatic neoplasms, potentially leading to misclassification and unnecessary surgical resection.

From a clinical perspective, accurate preoperative recognition of solid serous adenomas is important because these tumors are benign and may be managed conservatively in selected patients.^[3,10,11]^ However, in practice, differentiation from other solid pancreatic tumors remains challenging, and surgical resection is frequently performed when the diagnosis is uncertain, resulting in potential overtreatment of benign lesions.^[12–18,20,22,24,26]^ In this context, awareness of the broader imaging spectrum of solid serous adenomas, including atypical enhancement patterns such as those observed in the present case, may help refine the differential diagnosis and support more balanced clinical decision-making, particularly in avoiding unnecessary surgical intervention when imaging findings are atypical.

A limitation of this case is that a definitive preoperative diagnosis could not be established on the basis of imaging alone because of substantial overlap with other solid pancreatic neoplasms. In addition, as a single-case report, the imaging-pathologic correlation described here may not be generalizable to all cases of solid serous adenoma.

In summary, solid serous adenoma should be considered in the differential diagnosis of a solid-appearing pancreatic mass, particularly when the enhancement pattern is atypical and deviates from the more commonly reported hypervascular appearance. Recognition of this broader imaging spectrum, together with an understanding of its underlying histopathologic heterogeneity, may help improve diagnostic confidence, reduce misclassification as other solid pancreatic neoplasms, and ultimately avoid unnecessary surgical resection of benign pancreatic lesions.

## Acknowledgments

We would like to thank Editage (www.editage.co.kr) for English language editing.

## Author contributions

**Conceptualization:** Sohui Jin, Jisun Lee.

**Data curation:** Sohui Jin, Jisun Lee.

**Formal analysis:** Sohui Jin, Jisun Lee.

**Methodology:** Jisun Lee.

**Project administration:** Jisun Lee.

**Resources:** Sohui Jin.

**Supervision:** Jisun Lee, Bum Sang Cho, Kyung Sik Yi, Yook Kim, Chi-Hoon Choi, Chang Gok Woo.

**Visualization:** Sohui Jin.

**Writing – original draft:** Sohui Jin, Jisun Lee.

**Writing – review & editing:** Sohui Jin, Jisun Lee.
